# Fungal Species Diversity in French Bread Sourdoughs Made of Organic Wheat Flour

**DOI:** 10.3389/fmicb.2019.00201

**Published:** 2019-02-18

**Authors:** Charlotte Urien, Judith Legrand, Pierre Montalent, Serge Casaregola, Delphine Sicard

**Affiliations:** ^1^GQE-Le Moulon, INRA, Université Paris-Sud, CNRS, AgroParisTech, Université Paris-Saclay, Gif-sur-Yvette, France; ^2^Micalis Institute, INRA, AgroParisTech, CIRM-Levures, Université Paris-Saclay, Jouy-en-Josas, France; ^3^SPO, INRA, Montpellier SupAgro, Univ Montpellier, Montpellier, France

**Keywords:** microbial communities, metabarcoding, food microbial ecosystem, yeasts, *Kazachstania*, fermented food, bakers, farmer-bakers

## Abstract

Microbial communities are essential for the maintenance and functioning of ecosystems, including fermented food ecosystems. The analysis of food microbial communities is mainly focused on lactic acid bacteria (LAB), while yeast diversity is less understood. Here, we describe the fungal diversity of a typical food fermented product, sourdough bread. The species diversity of 14 sourdoughs collected from bakeries located all over France was analyzed. Bakeries were chosen to represent diverse bakery practices and included bakers and farmer-bakers. Both non-culture-based (pyrosequencing of Internal Transcribed Spacer 1 amplicons) and culture-based methods were used. While both identification methods were in agreement regarding the dominant yeast species of each sourdough, the ITS1 metabarcoding analysis identified an increased number of fungal species in sourdough communities. Two third of the identified sequences obtained from sourdoughs were *Saccharomycetales*, mostly in the *Kazachstania* genus. No *Saccharomycetales* species was shared by all the sourdoughs, whereas five other fungal species, mainly known plant pathogens, were found in all sourdoughs. Interestingly, *Saccharomyces cerevisiae*, known as “baker’s yeast,” was identified as the dominant species in only one sourdough. By contrast, five *Kazachstania* species were identified as the dominant sourdough species, including one recently described *Kazachstania* species, *Kazachstania saulgeensis* and an undescribed *Kazachstania* sp. Sourdoughs from farmer-bakers harbored *Kazachstania bulderi, Kazachstania unispora* and two newly described *Kazachstania* species, while sourdough from bakers mostly carried *Kazachstania humilis* as the dominant species. Such yeast diversity has not been found in sourdoughs before, highlighting the need to maintain different traditional food practices to conserve microbial diversity.

## Introduction

Microbes colonize all types of environments and are necessary for the function of all ecosystems, including human domesticated environments. They are commonly used for the fermentation of a wide range of primary products as diverse as milk, cereals, legumes, plant roots, vegetables, fish, and meat (for a review see Tamang et al., 2016). They play key roles in food texture and flavor (see for bread, Martinez-Anaya et al., 1990; Damiani et al., 1996; Hansen and Schieberle, 2005; Csoma et al., 2010; Birch et al., 2013) as well as in bio-preservation and nutritional compounds (Caplice and Fitzgerald, 1999; Bourdichon et al., 2012; Gobbetti et al., 2014, 2018; Gänzle and Ripari, 2016). The microbial community involved in the fermentation process include lactic acid bacteria (LAB) as well as fungi, particularly yeasts (reviewed in De Vuyst et al., 2014: De Vuyst et al., 2016; Carbonetto et al., 2018).

Microbial communities of fermented food products have long been studied using culture-based microbiological methods, in which a restricted number of cultivable clones are isolated after growth on different media, allowing the detection of only a fraction of the microbial population. In the last decade, metabarcoding approach, based on the sequencing of bacteria or eukaryotic markers from environmental DNA, became highly popular (reviewed in van Hijum et al., 2013; Kergourlay et al., 2015; De Filippis et al., 2017). This approach first focused on bacterial species and was more recently used to study fungi (reviewed in De Filippis et al., 2017). As a result, the number of publications describing fungal diversity using metabarcoding analysis remains lower than those describing bacteria (De Filippis et al., 2017). Fungal metabarcoding analysis of fermented products mostly focused on dairy products and alcoholic beverages (De Filippis et al., 2017). The analysis of fungal communities of plant-fermented food is less numerous but includes the cacao bean (Illeghems et al., 2012), the green olive (Arroyo-López et al., 2016), douchi (Yang et al., 2016), pu-erh tea (Zhang et al., 2016), kombucha (Reva et al., 2015; Coton et al., 2017), paocai (Liang et al., 2018), and sourdough (Minervini et al., 2015). The internal transcribed spacers (ITS1 and ITS2), including two non-coding parts of the ribosomal DNA unit situated between the small subunit (SSU) and the large subunit (LSU) rRNA genes, are the most frequently used fungal barcodes (Schoch et al., 2012; Vu et al., 2016, 2019). These barcodes have been recognized as a primary barcode in fungi and appears to better discriminate yeast species compared to the D1/D2 domains of the LSU region (Martin and Rygiewicz, 2005; Schoch et al., 2012; Vu et al., 2016). Amplification of either ITS1 or ITS2 was shown to be informative in describing fungal community composition. However, the analysis of fungal diversity is more difficult compared to the bacterial diversity for a variety of reason. First, the D1/D2 domains might be too much conserved to discriminate species. The ITS sequences are better to discriminate species but they harbor size polymorphisms, which render sequence alignment and comparison more tricky. Second, some ITS sequences are so small that they can be lost in the process of amplification and library making. Third, the ITS databases are either curated but not exhaustive (such as UNITE) or present a lot of redundancy (such as NCBI).

Among plant-fermented food, bread is of importance both from a cultural and a nutritive point of view. Leavened bread can be made using either yeast starter or using sourdough. Three types of sourdoughs are defined. Type I sourdough is a mixture of flour, water, bacteria and yeasts. It is a naturally fermented sourdough and is the traditional sourdough. Type II and type III sourdoughs are industrial types of sourdough. Usually, type I sourdough contains one dominant LAB species and one dominant yeast species, as well as a few other minor microbial species (Lhomme et al., 2015a, 2016; Minervini et al., 2015; Zhang et al., 2011, 2015; Dertli et al., 2016; Liu et al., 2016, 2018; Michel et al., 2016; De Vuyst et al., 2017). According to the literature, the most frequent LAB dominant species of sourdough are *Lactobacillus sanfranciscensis, L. brevis* or *L. plantarum*, and the most frequent yeast species are *Saccharomyces cerevisiae, Kazachstania humilis* (Jacques et al., 2016), *Pichia kudriavzevii, Torulaspora delbruecki*, and *Wickerhamomyces anomalus* (reviewed in De Vuyst et al., 2017; Carbonetto et al., 2018). Bacterial species diversity appears to be shaped by several drivers, including the bakery environment and technical processes (Scheirlinck et al., 2007; Minervini et al., 2014, 2015, 2018; reviewed in Gobbetti et al., 2016; Van Kerrebroeck et al., 2017). While the factors driving bacterial diversity have been studied, those shaping the distribution of yeasts remain poorly explored.

In Europe, the microbial species diversity of wheat sourdoughs has been well described in Italy (Corsetti et al., 2001; Foschino et al., 2004; Minervini et al., 2011; Gobbetti and Gänzle, 2012; Lattanzi et al., 2013; Minervini et al., 2014, 2015; Pontonio et al., 2016) and Belgium (Scheirlinck et al., 2007, 2009; Vrancken et al., 2010). Although bread is a traditional food product in France, few studies have analyzed sourdoughs’ microbial diversity (Lhomme et al., 2015a,b, 2016; Michel et al., 2016). In France, bread is often made from wheat either by farmer-bakers or by bakers. The dedicated term “farmer-baker” describe someone involved in both farming and bread production (Demeulenaere and Bonneuil, 2010).

Bread making practices are diverse, especially among bakeries that make bread with organic flour. Previous culture and non-culture-based analysis of bacterial species diversity in French sourdoughs revealed a low level of species diversity, even among sourdoughs made with organic flour and with different bread-making practices (Lhomme et al., 2015b, 2016; Michel et al., 2016). *Lactobacillus sanfranciscensis* was found as the main dominant species. The objective of this study was to describe the pattern of fungal species diversity in a collection of type I sourdoughs made with organic flour coming from bakeries with different bread-making practices and from different French regions using culture- and non-culture-based methods.

## Materials and Methods

### Field Survey and Sourdough Collection

A total of 14 bakers located in different French regions participated to this study. The study was carried out jointly by two French researcher teams. One worked on bacterial species, the other one on yeasts. The data on bacterial species diversity and biochemical properties of sourdoughs and breads have been previously published (Lhomme et al., 2015b). We analyzed the yeast species diversity as well as the bread-making practices.

All bakers produce bread in a low-input agro-food ecosystem. They explained their bread-making practices during diverse interviews. The output of these interviews is presented in the results section and provides details on the specific practices of the bakers we studied. Broadly, each bread making process starts from the chief sourdough, which comes either from a piece of dough or a piece of final sourdough from the previous bread-making process. Water and flour are added to the chief sourdough to constitute the final sourdough. This is called feeding or back-slopping. The flour is obtained by milling ancient and/or modern wheat varieties using cylinders or stone mills. Once the final sourdough is reactivated, kneading can start by mixing the final sourdough with flour, water, and salt. In some cases, the baker also adds commercial baker’s yeast. After kneading, the first fermentation lasts for a variable amount of time, depending on bakers. The dough is then divided and shaped. A second fermentation occurs before cooking. Bread is cooked in wood-fired or electric ovens. From one bread making process to the next, the chief sourdough is stored either at room temperature or in the refrigerator. During this process, thirteen well-documented characteristics were retained from the interview to reflect the diversity of bakery practices (Table 1).

**Table 1:** Diversity of bread making practices among 14 bakers located in France.

Baker	Region	Baker Status	Wheat	Mill	Flour origin	Water origin	Age of SD	Origin of Chief SD	Temp. of chief SD	Knead-ing	% of SD in the dough	No. of BM	No. of feedings between BM	Total no. of feeding per week	Baker yeast
1	Poitou Charentes	FB	Mix	Stone	Own	Tap	12	SD	AT	Mech.	43	2	2	4	No
2	Aquitaine	FB	Anc.	Stone	Own	Tap	0.5	D	Cold	Mech.	16	2	3	6	No
3	Rhone-Alpes	FB	Anc.	Stone	Own	Tap	10	SD	Cold	Manual	15	3	2	6	No
4	Pays de Loire	B	Mix	Stone	Miller	Tap	20	D	Cold	Mech.	33	4	1	4	No
5	Ile de France	B	NA	Stone	Miller	Tap	20	SD	Cold	Mech.	10	6	1	6	Yes
6	Champagne Ardenne	B	Mod.	Stone	Miller	Tap	1	D &SD	Cold	Mech.	17	6	1	6	No
7	Bretagne	B	NA	Stone	Miller	Filtered	29	D	Cold	Mech.	9	4	1	4	No
8	Pays de Loire	B	NA	Stone	Miller	Filtered	33	SD	AT	Mech.	24	7	1	12	Yes
9	Ile de France	B	Mod.	Stone	Own	Tap	2	D &SD	AT	Mech.	18	5	1	5	Yes
10	Provence Alpes Côte d’Azur	B	NA	Cylind.	Miller	Tap	13	SD	Cold	Mech.	20	6	2	12	No
11	Bourgogne	B	NA	Stone	Miller	Tap	NA	SD	Cold	Mech.	4	7	4	28	Yes
12	Bretagne	FB	Anc.	Stone	Own	Filtered	NA	D	AT	Manual	14	2	2	4	No
13	Franche-Comté	B	Anc.	Stone	Miller	Tap	0.5	D	Cold	Manual	11	1	3	3	No
14	Alsace	B	Mod.	Stone	Miller	Filtered	15	D	AT cold	Mech.	2	4	2	8	No

*The bakers were either farmer-baker (FB) or baker (B). They all use wheat from low-input farming for making their sourdough. A total of 13 characteristics during the process are documented. Ancient (Anc.) or modern (Mod.) wheat varieties were milled with either millstones (Stone) or cylinder (Cylind) mills. The flour was made by the baker (own) or by another miller (miller). The water was either coming from tap or was filtered. The sourdough age in year since the first mix of water and flour was also reported. The chief sourdough came either from the final sourdough (SD) or from the dough (D) of the last bread making process and was stored at ambient (AT) or cold temperature (Cold). The kneading was either mechanical (mech.) or manual. The percent of final sourdough was measured as the proportion of sourdough relative to the mass of dough. The number of bread-making process (BM) per week was reported, as well as the number of feeding between bread-making process and per week. In four cases (yes), commercial baker yeast was used regularly for making other bakery’s products and in one case it was also added to make bread (Baker 5).*

In each bakery, the final sourdough of one bread-making run was sampled twice. One sample was taken from the surface of the sourdough and the other from the center. All samples were kept at 4°C for 3–7 or sent by post-mail at ambient temperature and then kept cold before the culture-based analysis was initiated. Yeasts were first isolated and identified by the culture-based technique, while part of the sourdough was kept at -20°C for the non-culture-based analysis.

### Culture-Based Method of Yeast Species Identification

Yeast enumeration, isolation and DNA extraction were performed according to Lhomme et al. (2016).

Two pieces of sourdough (one was taken from the surface of the sourdough and the other from the center) were diluted independently and sowed on different petri dishes. From 39 to 40 isolates per sourdough were then cloned, stored and further analyzed, except for sourdough 14, from which only 12 isolates were characterized. Each cloned isolate represents a strain. The strains were isolated by randomly picking colonies on different petri dishes.

With 40 strains analyzed, the probability of detection of one species representing 10% of the sourdough species is over 98% but falls at 65% when only 10 strains are analyzed.

For DNA extraction, strains were grown at 30°C in 1 ml of YE (1% YE, 2% glucose) shaken at 200 rpm. After digestion of the pellet for 1 h at 37°C using 10 units of zymolyase (Euromedex, Souffelweyersheim, France) in 0.5 mL of sorbitol 1 M, Na2EDTA 0.1 M, pH 7.5 buffer, a standard method of DNA extraction was used (Lhomme et al., 2016). All the 519 yeast isolates were identified on the basis of their pattern of *AluI* digestion of the amplified rDNA Non-Transcribed Spacer 2 (NTS2) (Nguyen and Gaillardin, 1997). Species identification was confirmed by sequencing the D1/D2 rDNA region (Kurtzman and Robnett, 2003) for 60 strains representatives of the different *AluI* profiles and by sequencing the actin coding gene, *ACT1* for 46 strains (Daniel and Meyer, 2003). All the oligonucleotide PCR primers used were obtained from Invitrogen (Invitrogen, Cergy Pontoise, France) and PCR products were Sanger sequenced on both strands by Eurofins (Supplementary Table S1).

DNA sequence alignments were carried out using the pairwise alignment method for the forward and reverse complement sequences of each strain in order to obtain a consensus sequence. Identification queries were fulfilled by a BLAST search of the National Center for Biotechnology Information database (NCBI, Bethesda, United States) and the YeastIP databases^1^. A similarity of more than 99% to gene sequences of type strains was used as the criterion for species identification.

### Non-culture Based Method of Fungal Identification

#### Synthetic Sourdough

As control for pyrosequencing analysis, we created a synthetic sourdough. One representative strain of nine species (*Candida carpophila, Hyphopicchia pseudoburtonii, Kazachstania humilis, Kazachstania bulderi, Kazachstania saulgeensis, Kazachstania unispora, Rhodotorula mucilaginosa, Saccharomyces cerevisiae, Torulaspora delbrueckii)* previously isolated from French organic sourdoughs (Jacques et al., 2016; Lhomme et al., 2016) was included in the synthetic sourdough (Supplementary Table S2). After 12 h in 5 mL of YE media at 30°C, cell counts for each strain were determined using a Coulter counter (Beckman Coulter, Brea, CA United States). All strains were mixed at the same density (10^7^ UFC/g of sourdough) together with organic wheat flour (0.76 g, organic T110 wheat flour from Biocer) and distilled water (1 g). This synthetic sourdough, was then stored at -20°C for 10 days to mimic the storage process of bakeries sourdough samples. No yeast growth phase in the sourdough was included before storage to avoid adding a factor leading to inequality between yeast density.

#### Total DNA Extraction

The DNA extraction was carried out on a sample, taken independently from the ones used for the culture-dependent analysis. For each sourdough, 200 mg of sourdough was used to extract DNA with the PowerSoil DNA Isolation Kit (MO BIO laboratories, Inc., Carlsbad, CA, United States)).

To improve the yield, the following changes were made to the manufacturer’s extraction protocol: after adding C1 solution to an aliquot of sourdough in the bead tubes, the tubes were incubated at 65°C for 10 min and then horizontal mixed at 30 Hz for 5 min. The tubes were then put on ice before continuing with the kit protocol. The final elution step with C6 solution was carried out twice with the addition of 40 μL (total 80 μL).

#### Amplicon Preparation for Pyrosequencing

##### Choice of primer pairs

Pyrosequencing was carried out on the ITS1 rDNA region. Among 29 ITS1 primers published in the literature (Supplementary Table S3), we selected the ITS primers that could specifically amplify fungal DNA but not plant DNA. To do so, we first built an ITS sequences database by blasting the S288C *Saccharomyces cerevisiae* ITS1-5.8S-ITS2 sequence against the NCBI non-redundant nucleotide database, including any sequences with both an e-value < 10^-4^ and a > 80% identity. We also added *Poaceae* ITS sequences. Overall, there were 1995 sequences in this database. Second, we blasted 29 primers from the literature (Supplementary Table S3) on this database (parameters: e-value < 10^-2^; > 80% identity). From this analysis, six primers theoretically allowed amplification of fungal but not *Poaceae* ITS regions: forward primers NSI1 (Gardes and Bruns, 1993) and NSA3 (Martin and Rygiewicz, 2005), and reverse primers ITS4 and NLB4 (Gardes and Bruns, 1993), 58A2R and NLC2 (Martin and Rygiewicz, 2005). Two of these hypothetical fungal-specific primer pairs (NSA3-NLC2 and NSA3-58A2R) were tested on DNA extracted from 17 natural sourdough yeast strains representing the ten species previously identified in natural French organic sourdoughs (Jacques et al., 2016; Lhomme et al., 2016) and from three plant species: wheat, maize and nigella (Supplementary Table S2). The primers pair, NSA3 (5′–AAACTCTGTCGTGCTGGGGATA–3′) and 58A2R (5′–CTGCGTTCTTCATCGAT–3′) (Martin and Rygiewicz, 2005) gave fungal specific amplification and was selected for ITS pyrosequencing.

##### Amplicon preparation

Amplicons for pyrosequencing were prepared in two steps: first, ITS1 amplification and second, ITS1 amplification with barcode addition. ITS1 region was amplified from 100 to 200 ng of total sourdough DNA incubated in a 50 μL of the following mix reaction: 0.2 mM of each dNTP, 1 mM of MgCl_2_, 15 μM of each primer, 2.6 unit of *Taq* Polymerase (Roche Expand High Fidelity, Roche, Mannheim, Germany), 1X Roche Expand High Fidelity buffer. Two consecutive amplification regimes were performed for the first ITS1 amplification step. After an initial denaturation at 95°C for 2 min, 10 cycles of amplification were conducted as follows: denaturation at 95°C for 15 s, annealing at 60°C for 30 s, and extension at 72 °C for 45 s. The final extension was at 72°C for 45 s. A second 10 cycles of amplification was performed: denaturation at 95°C for 15 s, annealing at 60°C for 30 s, and extension at 72°C for 45 s with addition of 5 s extension time at each subsequent cycle. The final extension was at 72°C for 7 min.

Addition of specific barcodes was performed in 50 μL reactions containing 2 μL of the previous PCR product, 0.2 mM of each dNTP, 0.5 mM of MgCl_2_, 0.3 μM of each specific primer containing the specific index and the Eurofins MIDs (Supplementary Table S4), 2.6U de Taq Expand High Fidelity of Roche and 1X Buffer Expand High Fidelity. After an initial denaturation at 95°C for 2 min, 5 cycles of amplification were conducted as follows: denaturation at 95°C for 15 s, annealing at 60°C for 30 s, and extension at 72°C for 45 s. The final extension was at 72°C for 7min.

Each sample was column purified with the MinElute PCR Purification Kit (Qiagen, 28006), followed by concentration with a speed vacuum (Savant SPD IIIV Speed Vac Concentrator, Thermo Electron Corporation) for 20 min to obtain 60 ng/μL as measured by NanoDrop (NanoDrop 2000 UV-Vis Spectrophotometer, Thermo Fisher Scientific). Finally, amplicons were sent to Eurofins^2^ for the titanium FLX pyrosequencing (Roche technology, compared with other new-generation sequencing systems in Liu et al., 2012).

#### Metabarcoding Data Analysis

The metabarcoding data were processed with the QIIME (Quantitative Insights Into Microbial Ecology) v 1.8.0 (Caporaso et al., 2010) pipeline.

##### Database

UNITE fungal ITS database version 7 (17,421 sequences; downloaded at http://unite.ut.eerelease_01.08.2015, Kõljalg et al., 2013) was completed with 13 ITS1 Sanger sequences obtained from five natural sourdough strains and eight type strain sequences (Supplementary Table S5) corresponding to 11 previously described bread sourdough species. The additional sequences were non-redundant with the downloaded UNITE database sequences. The UNITE database completed with the sourdough strains contains 432 *Saccharomycetes* reference sequences. Hereafter in this article, we term the downloaded UNITE database “UNITE only” and the UNITE database completed with the sourdough strain sequences “UNITE completed.”

##### Filtering, quality trimming and clustering into OTU

To conserve high quality sequences only, we trimmed and filtered the reads. For filtering, raw pyrosequencing fna and quality files were transformed to fastq files. Reads were truncated with the PRINSEQ tool at the 3′ position at the quality threshold of 34, and any read <127 bp was discarded. The following steps were performed using QIIME: chimera detection using usearch61 against the uchime_reference dataset^3^; operational taxonomic unit (OTU) selection using uclust (Edgar, 2010) with the open reference OTU-picking workflow and a threshold of 99% pairwise identity; and taxonomy assignment using the RDP Classifier 2.2 (Wang et al., 2007) and using the RDP Classifier 2.2 (Wang et al., 2007) and BLAST against the “UNITE completed” database.

Assignment was performed on the most representative sequence of each OTU according to the “UNITE completed” database (17,434 sequences) and cut in the SSU region at the sequence CGTAAGGT (Supplementary Table S5) so as not to bias assignment in favor of the SSU region, which is well conserved between species.

### Data Analysis

The datasets are available under the URL http://doi.org/10.5281/zenodo.1472295, under a creative commons attribution.

The results regarding Sourdough 14 gave peculiar data (see section Identification of Yeast Species in Sourdough by the Culture-Based Method) and was thus excluded from most of the analysis described below. Indeed, few microbial isolates could be retrieved from the sourdough. In addition, metabarcoding data revealed only two reads assigned to saccharomycete species over 8444, while this class is supposed to contain the dominant sourdough yeast flora.

#### Analysis of Bread-Making Practices

To describe the diversity of bakery practices, we performed a multiple correspondence analysis (MCA) on 13 bread-making process characteristics described for each baker (Table 1). Bakers were either farmer-bakers (producing their own wheat grains) or bakers (only making bread). This “status” variable was not used to build the MCA axis and was only used as a Supplementary variable. To avoid classes with only one observation, we recoded variables as follows: conservation at “cold & room temperature” were included in the “cold” group; the modalities “dough + SD” and “dough” of the sourdough origin variable were grouped together in the “dough” modality; the total number of feedings per week was recoded as “frequent” if it exceeded 6 or “rare” otherwise; the number of bread makings per week was recoded as “frequent” (>3) or “rare”; the fraction of sourdough in the dough was recoded as “low” (<20%) or “high”; the sourdough age was recoded as “recent” (2 years or under), “average” (between 2 and 20 years), or “old” (>20 years); and the number of feedings between bread makings was recoded as “1,” “2,” and “3+4”. For each variable, missing values were treated as a modality and coded “UN”. Then, using the first two axes of the MCA, we clustered the bakeries into groups using a k-means algorithm and used this “practice type” variable in the following sections.

#### Analysis of Microbial Species Diversity

The microbial species richness was characterized over all sourdough and fungal orders. The other alpha diversity as well as beta diversity indices were carried out only on *Saccharomycetales* species, as they may be involved in fermentation and they were the only species found using the culture-based method.

Diversity indices were only calculated on the metabarcoding data. Indeed, only 40 strains were analyzed by culture-based method which may bias estimates. For each sourdough, we computed the species richness as well as the Shannon and Simpson alpha diversity indices. The Shannon and Simpson indices value were also converted to effective number of species (Jost, 2006). Between-sample community dissimilarity was quantified by computing weighted UniFrac distances (Lozupone and Knight, 2005) using the Gunifrac R-package on the rarefied OTU abundance matrix. We used the OTU phylogenetic tree constructed with *Candida boleticola* species as the outgroup.

Based on the UniFrac distance matrix, the sourdoughs were clustered into groups using hierarchical clustering. The link between practices and community composition was studied by performing non-parametric analysis of variance on the Gunifrac distance matrix as well as exact Fisher tests between each practice variable and the group of sourdoughs clustered according to the UniFrac distance. To account for multiple testing, we adjusted the p-value with the false discovery rate (FDR) method (Benjamini and Hochberg, 1995).

#### Analysis of Sourdough Properties and Their Link With Bread-Making Practices

To study the relation between the microbial population densities and the biochemical properties of the sourdough, we performed a principal component analysis (PCA) on the average values of the yeast and LAB population densities (on a log10 scale), the pH, the lactic acid and acetic acid contents, the TTA (Lhomme et al., 2015b). Sourdough 14 was not used to build the PCA axes but used as a supplementary individual. To investigate the relationship between bread making practices, the community composition, and the population densities and biochemical properties of the sourdough, we used the bread-making practices groups built from the MCA and the community group as supplementary variables.

## Results

### Characterization of Bakery Practices

**FIGURE 1:**
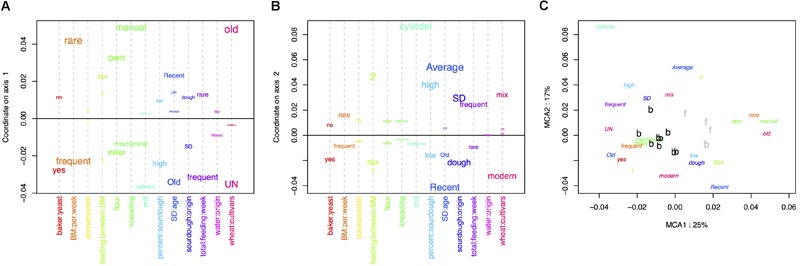
Multiple correspondence analysis (MCA) on the 13 characteristics of the bread-making process described for each baker **(A)** variables explaining the first axis, **(B)** variables explaining the second axis, **(C)** distribution of baker practices on the first two axis.

The MCA performed on the bakery practices is represented in Figure 1. The first axis (explaining 25% of the variance) opposed intensive practices (frequent bread-makings, frequent sourdough back-slopping, use of commercial yeast in the bakery, flour originating from a miller, mechanical kneading, unknown wheat cultivar) against extensive practices (manual kneading, use of own flour, rare bread-making processes and sourdough back-sloppings, use of ancient wheat populations) (Figure 1A). The second axis of the MCA was mostly explained by the characteristics of the sourdough (origin, age, percent in the dough) as well as the mill type (Figure 1B).

The clustering into two groups separated bakers having intensive practices from bakers having extensive practices (Figure 1C). All bakers but one were in the “intensive practices” group, and all farmer-bakers plus one baker were in the “extensive practices” group. Hereafter, we named these two groups “baker-like” and “farmer-baker-like” and encoded them “B” and “F,” respectively. We can note that this clustering was largely influenced by the number of bread making processes per week and match perfectly this variable. Increasing the number of groups to three increased the fraction of between-group variance from 50 to 73%. However, this would result in a group with only two bakers, which would make statistical analysis and interpretation difficult. For this reason, we decided to discard further interpretation of the second axis of the MCA and to keep the first axis clustering into two groups in the following analyses.

### Identification of Yeast Species in Sourdough by the Culture-Based Method

*Alu*I digestions of the NTS2 region were obtained on 519 yeast strains isolated from 13 natural organic sourdoughs. Eight yeast species were identified corresponding to seven *Alu*I digestion patterns (Table 2). One of them was recently discovered: *Kazachstania saulgeensis* was found in Baker 1’s sourdough and was called by the name of its town of origin, Saulgé (Jacques et al., 2016; Sarilar et al., 2017). Another profile corresponds to a yet undescribed *Kazachstania* species, found in Baker 2’s sourdough. The ITS sequence of this undescribed species, represented by strain B2_TP1_4 (sequence Accession Number KX458458.1), had 100% identity with the sequence of strain IMBRR1 (sequence Accession Number KC118125.1), isolated from Bulgarian boza, a cereal-based fermented beverage. Based on the rRNA sequence comparison, the most closely related *Kazachstania* species to strain *Kazachstania* sp. B2_TP1_4 is *Kazachstania exigua.* They share 96% identity in the ITS sequence and 99% identity in the 26S D1/D2 LSU rDNA sequence (large subunit rRNA gene) between the *K. exigua* neotype strain CBS379^NT^ and the strain B2_TP1_4. Based on the RPB2 coding gene sequence comparison, the most closely related *Kazachstania* species to strain *Kazachstania* sp. B2_TP1_4 (Sequence Accession Number LT158256.1) is *Kazachstania australis* CBS 194^T^ (Sequence Accession Number LT158259.1), with 93% identity followed by *K. exigua* CBS 379^NT^ (Sequence Accession Number LT158276.1) with 92% identity.

**Table 2:** Yeasts and bacterial species counts in 13 natural sourdoughs as studied by the culture-based method.

Sourdough	1	2	3	12	13	4	5	6	7	8	9	10	11	Total
Baking pratice group	F	F	F	F	F	B	B	B	B	B	B	B	B	
**Yeasts**							
*Saccharomyces cerevisiae*	1								1		40			42
*Kazachstania bulderi*			40	39		40								119
*Kazachstania barnettii*					40									40
*Kazachstania sp.*		11												11
*Kazachstania saulgeensis*	39													39
*Kazachstania unispora*		29												29
*Kazachtania humilis*							40	40	39	39		40	40	238
*Candida carpophila*										1				1
**Bacteria**							
*Lactobaciilus sanfranciscensis*	22	36	11	33	14	12	10	33	29	25	4	31	33	293
*Lactobaciilus sakei*						14								14
*Lactobaciilus paramimentarious*							11							11
*Lactobaciilus hammesii*							10							10
*Lactobaciilus plantarum*			5											5
*Lactobaciilus curvatus*					4									4
*Lactobaciilus pentosus*							2							2
Other											2			2

*The sourdoughs are characterized by the identity of the baker and the bread-making practice. The yeast species were identified in this study. The bacteria ones were obtained from a previously published paper (Lhomme et al., 2015b).*

Among the 519 strains (Table 2), 45.9% belonged to *K. humilis* and 22.9% belonged to *K. bulderi.* The remaining were *S. cerevisiae* (8.1%), *K. barnettii* (7.7%), *K. saulgeensis* (7.5%), *K. unispora* (5.6%), *Kazachstania* sp. (2.1%), and *Candida carpophila* (0.2%). No yeast species was shared by all 14 sourdoughs. *K. humilis* was the most widely distributed species. It was detected as a unique species in four sourdoughs and as the dominant species together with other yeast species in two sourdoughs. *Kazachstania bulderi* was found as a unique species in three sourdoughs. *S. cerevisiae* was identified as a unique species in one sourdough and as a minor species (1 isolate) in sourdoughs 1 and 7. The new species *K. saulgeensis* was dominant in sourdough 1, while the *Kazachstania* sp. strains described above (represented by B2_TP1_4) were found together with *Kazachstania unispora* strains in sourdough 2. It is important to note that, overall, only 42 (from sourdoughs 1, 7, and 9) out of 519 isolates belonged to the *S. cerevisiae* species that is usually used in commercial starters as a leavening agent in bakery products.

### Sourdough Fungal Composition Described by Non-culture-Based Analysis

#### Synthetic Sourdough

A synthetic sourdough (sourdough 15), made of flour and nine yeast species previously found to be present in French sourdoughs, was analyzed as a control for our method of pyrosequencing. The eight *Saccharomycetes* species (*Candida carpophila, Hyphopicchia pseudoburtonii, Kazachstania humilis, Kazachstania bulderi, Kazachstania saulgeensis, Kazachstania unispora, Saccharomyces cerevisiae, Torulaspora delbrueckii)* as well as the single *Basidiomycota* species (*Rhodotorula mucilaginosa)* mixed in equal density in the synthetic sourdough were all detected but in different proportions, from 6 reads for *C. carpophila* to 296 reads for *K. humilis*. In addition, the synthetic sourdough contained other yeast species, which probably came from the flour. Overall, it contained 40.31% *Dothideomycetes* class, mainly represented by the *Capnodiales* order (31.8% of the total synthetic sourdough reads) and 14.59% *Sordariomycetes* class, including the *Hypocreales* and *Xylariales* orders (7.51 and 7.01% of the total synthetic sourdough reads, respectively). The *Basidiomycota* phylum represented 18.37% of the reads. Surprisingly, the *Saccharomycetales* order represented only 9.85% of the reads (972 reads), suggesting that the *Saccharomycetales* were not dominant in the flour and not well extracted from the yeasts added to the synthetic sourdough.

#### Natural Sourdoughs

A total of 689 OTUs corresponding to 202,225 reads were selected for analysis because they were represented by more than five reads after chimera detection. Each sourdough sample contained between 104 (Sourdough 10) and 351 (Sourdough 7) OTUs (Supplementary Table S6).

The assignment was done using either RDP or BLAST after completing the UNITE database with ITS sequences obtained from sourdough strains (Table 3). Database completion improved the rate of OTU assignment at the species level (data not shown). Most reads were assigned to *Ascomycota* (99.0% with RDP, 94.2% with BLAST) while *Basidiomycota* (0.2% with RDP, 1% with BLAST) and *Anthophyta* (0.1% with RDP, none with BLAST) were much less represented. A total of 4.7% (with BLAST) and 0.7% (with RDP) of the reads remained unidentified at the phylum level. Overall, at the species level, BLAST was able to assign more OTUs then RDP (Table 3). It allowed the detection of 32 species that RDP did not. For this reason, only BLAST assignments are presented below.

**Table 3:** Number of reads assigned to species level by BLAST and RDP.

Phylum	Order	Species	RDP	BLAST
*Ascomycota*	*Capnodiales*	*Davidiella tassiana*	269	14,065
		*Capnobotryella* sp. *MA 4775*	11	34
	*Dothideales*	*Aureobasidium pullulans*	0	285
	*Pleosporales*	*Didymella exigua*	0	32
		*Sclerostagonospora phragmiticola*	0	11
		*Bipolaris maydis*	0	31
		*Chalastospora ellipsoidea*	0	24
		*Pleospora herbarum*	0	541
		*Pyrenophora tritici_repentis*	313	319
	*Eurotiales*	*Aspergillus caesiellus*	17	17
		*Aspergillus cibarius*	204	5,435
		*Aspergillus flavus*	1	1
		*Aspergillus penicillioides*	11	11
		*Aspergillus subversicolor*	29	29
		*Penicillium polonicum*	10	10
	*Helotiales*	*Botrytis caroliniana*	0	149
	*Saccharomycetales*	*Candida boleticola*	8	8
		*Candida carpophila*	0	14
		*Kazachstania humilis*	69,806	69,898
		*Debaryomyces udenii*	0	8
		*Hyphopichia pseudoburtonii*	0	29
		*Kazachstania barnettii*	12,755	12,755
		*Kazachstania bulderi*	24,494	24,494
		*Kazachstania exigua*	0	1,369
		*Kazachstania saulgeensis*	11,869	11,869
		*Kazachstania servazzii*	24	24
		*Kazachstania unispora*	5,749	5,749
		*Saccharomyces cerevisiae*	2,046	2,046
		*Torulaspora delbrueckii*	19	19
	*Diaporthales*	*Diaporthe australafricana*	0	46
		*Diaporthe citrichinensis*	0	7
		*Phomopsis sp CLH1_10*	6	6
	*Glomerellales*	*Arthrinium sacchari*	0	34
		*Claviceps purpurea var phalaridis*	139	780
		*Sarocladium strictum*	0	45
		*Fusarium tricinctum*	0	974
	*Hypocreales*	*Gibberella zeae*	0	3,504
	*Incertae sedis*	*Plectosphaerella sp CCF3811*	19,861	1,096
	*Xylariales*	*Microdochium bolleyi*	0	8
		*Monographella nivalis*	0	3,604
*Basidiomycota*	*Incertae sedis*	*Tilletiopsis minor*	0	17
	*Tilletiales*	*Tilletia controversa*	183	183
	*Sporidiobolales*	*Rhodotorula mucilaginosa*	0	3
		*Sporobolomyces ruberrimus*	0	9
	*Cystofilobasidiales*	*Itersonilia perplexans*	0	6
		*Udeniomyces pannonicus*	0	55
	*Tremellales*	*Bandoniozyma aquatica*	0	8
		*Bullera globospora*	0	26
		*Bullera unica*	0	26
		*Cryptococcus heimaeyensis*	0	7
		*Cryptococcus laurentii*	0	95
		*Cryptococcus sp SM13L02*	0	21
		*Cryptococcus victoriae*	9	588
		*Cryptococcus wieringae*	0	220
		*Dioszegia fristingensis*	0	4
		*Dioszegia hungarica*	0	55
	*Trichosporonales*	*Trichosporon cutaneum*	0	6
	*Ustilaginales*	*Pseudozyma flocculosa*	0	18
		*Sporisorium panici_leucophaei*	0	80
	*Wallemiales*	*Wallemia sebi*	0	90
		total	147,833	160,897

**FIGURE 2:**
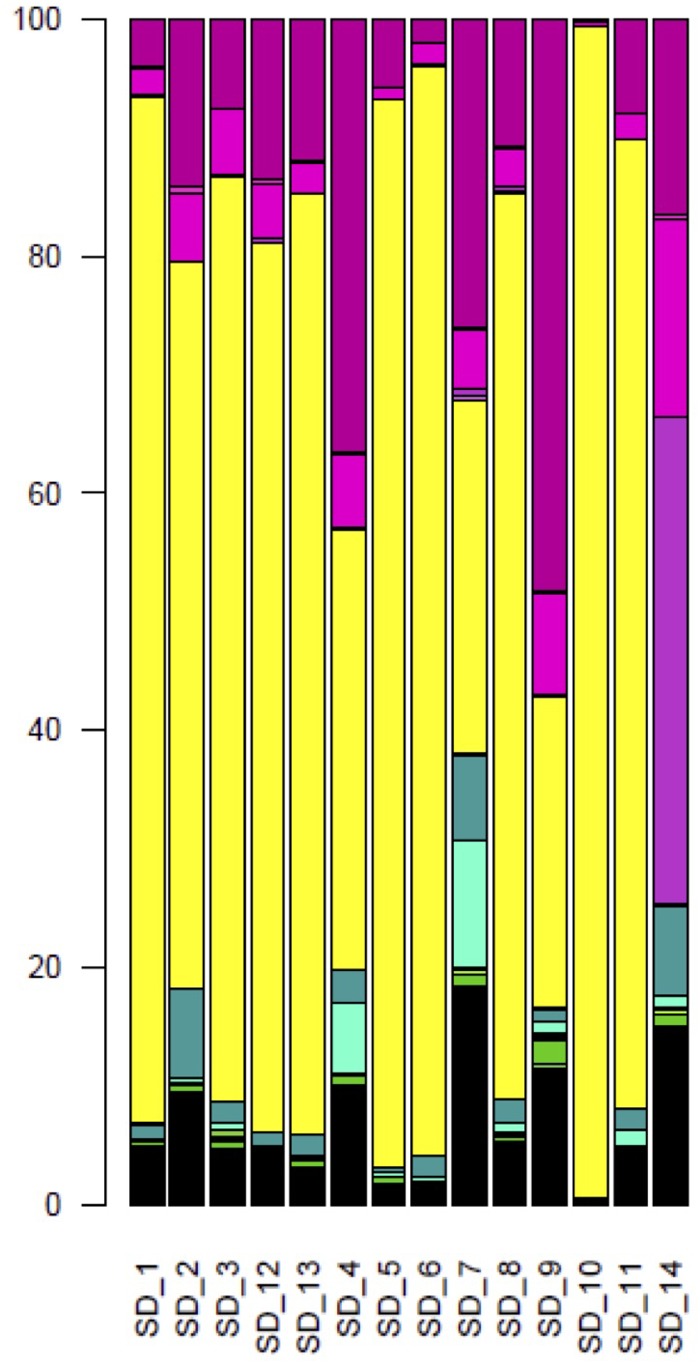
Composition of fungal orders, after BLAST assignments, for each natural sourdough. In yellow, *Saccharomycetales*; in medium violet red, *Capnodiales*; in hot magenta, *Pleosporales*; in fuchsia, *Eurotiales*; in Persian green, *Hypocreales*; in bright turquoise, *Xylariales*; in free speech green, *Tremellales*; in razzle dazzle rose, *Dothideales*; in apple, *Tilletiales*; in green yellow, *Filobasidiales*; in orchid, *Helotiales*; in La Palma, *Sporidiobolales*; in lime, *Wallemiales*; in fern, *Ustilaginales*; in turquoise blue, *Diaporthales*; in Kelly green, *Cystofilobasidiales*; in Pacific blue, *Glomerellales*; in myrtle, *Trichosporonales*; in bright green, *Exobasidiales*.

Over all sourdoughs, the *Saccharomycetes* class represented 66.7% (128,282) of the reads (Supplementary Table S6). *Dothideomycetes* was the second most represented class, with 18.1% of the reads. The other classes were represented by less than 5% of the reads. With the exception of Sourdough 14, the *Saccharomycetales* order was the most common in each natural sourdough, representing 26% (1,890 reads out of 7212 reads in Sourdough 9) to 99% (13,569 reads out of 13723 reads in Sourdough 10) of the reads (Figure 2). Other orders were also found : the *Capnodiales* order represented from 3% (43 reads, Sourdough 10) to 48% (3,481 reads, Sourdough 9) of reads in each sourdough; *Pleosporales* from 0.01% (35 reads, Sourdough 10) to 0.7% (130 reads in Sourdough 7); *Eurotiales* from 0% (0 reads, Sourdough 1 and 13) to less than 0.6% in the other sourdoughs (less than 100 reads per sourdough); *Hypocreales* from 0.1% (14 reads, Sourdough 10) to 8% (889 reads, Sourdough 2); and *Xylariales* from less than 0.1% (2 reads, sourdough 10) to 11% (1,929 reads, Sourdough 7) (Figure 2).

At the species level, a total of 160,897 reads (79.6% of the total number of reads) were assigned to 60 species (Supplementary Table S7). Overall, only 20.3% of the reads were assigned to non-*Saccharomycetales* species. Five non-*Saccharomycetales* species, known plant pathogens, were identified in all sourdoughs: *Davidiella tassiana* (*Capnodiales* order), *Claviceps purpurea* (*Hypocreales*), *Fusarium poae* (*Hypocreales*), *Monographella nivalis* (*Xylariales*) and *Cryptococcus victoriae* (*Filobasidiales*). However, these were identified at less than 1% of each sample in Sourdoughs 1 to 13. By contrast, most of the reads (79.7%) were assigned to the *Saccharomycetales* species, with 13 species detected. Over these 13 species, eight were also detected by culture-based method, including seven dominant species (*K. humilis, K. bulderi, K. barnettii, Kazachstania sp*., *K. saulgeensis, K. unispora, S. cerevisiae*) and one rare species (*Candida carpophila*). The *K. humilis* species was the most frequent species and was found in 12 out of 14 sourdoughs. In five sourdoughs, this species was dominant and was represented by more than 50% of the sourdough reads. In Sourdough 7, this species was represented by 30% of the reads, but this sourdough also had the highest number of unassigned reads. Finally, in six sourdoughs, this species was represented by less than 1% of the reads. The species *K. bulderi* was also recurrently found at high frequency in three sourdoughs (from 38 to 77.8% of the sourdough reads) and was detected at low frequency (below 2%) in five sourdoughs. Several other *Kazachstania* species were detected as dominant species in a single sourdough. This was the case for *K. barnettii* (79.1% of the reads in sourdough 13), *K. saulgeensis* (86.3% of the reads in sourdough 1), and *K. unispora* (48.8% of the reads in sourdough 2). The species *K. servazzii*, previously found in French sourdough as dominant species (Lhomme et al., 2015a), was detected twice but only at a frequency below 0.2%. Interestingly, the species *S. cerevisiae* was detected as the dominant species only once (25.6% of the reads in sourdough 9) although it was found at a frequency below 1% in nine other sourdoughs.

### Pattern of Fungal Species Diversity Among Sourdoughs

The within-sourdough yeast species diversity (alpha diversity) (Table 4) and the between-sourdough yeast species diversity (beta-diversity) was analyzed using metabarcoding data only since it allows a deeper analysis of the fungal community. Only the reads identified as *Saccharomycetales* were retained (Supplementary Table S7), as these species may be involved in fermentation. Sourdough 14 was discarded from this analysis, as only 2 reads were identified as *Saccharomycetales* in this sourdough.

**Table 4:** Number of reads assigned to each species of the *Saccharomycetale* order and alpha diversity in 13 natural sourdoughs as studied by pyrosequencing.

Sourdough	1	2	3	12	13	4	5	6	7	8	9	10	11		Total no. of reads in baker’s sourdoughs
Baking pratice’s group	F	F	F	F	F	B	B	B	B	B	B	B	B	Synthetic sourdough	
*Saccharomyces cerevisiae*	27	0	3	6	1	0	58	0	1	50	1845	46	9	86	2046
*Kazachstania barnettii*	2	0	1	13	12420	0	120	0	0	164	34	1	0	0	12755
*Kazachstania bulderi*	0	1	9498	9724	0	4974	0	0	2	290	1	0	4	221	24494
*Kazachstania exigua*	0	1357	0	2	0	0	0	0	0	10	0	0	0	0	1369
*Kazachstania humilis*	25	87	11	69	0	23	9760	17988	5424	12227	5	13499	10780	292	69898
*Kazachstania saulgeensis*	11739	0	0	17	0	0	0	0	1	111	0	0	1	52	11869
*Kazachstania servazzii*	0	1	0	0	0	0	0	0	0	0	0	23	0	0	24
*Kazachstania unispora*	1	5681	0	6	0	0	0	0	2	59	0	0	0	19	5749
*Torulaspora delbrueckii*	1	0	0	0	16	0	0	0	1	1	0	0	0	16	19
*Candida boleticola*	0	0	0	0	0	0	0	0	7	1	0	0	0	0	8
*Candida carpophila*	0	0	0	0	0	0	0	1	0	13	0	0	0	6	14
*Debaryomyces udenii*	0	0	0	0	0	0	0	0	7	1	0	0	0	0	8
*Hyphopichia pseudoburtonii*	0	0	0	3	7	0	0	0	1	9	5	0	2	280	27
Richness	6	5	4	8	4	2	3	2	9	12	5	4	5	8	
Simpson	0.0105	0.3292	0.0040	0.0250	0.0045	0.0100	0.0359	0.0003	0.0113	0.1075	0.0517	0.0109	0.0039		
Simpson true diversity	1.01	1.49	1.00	1.03	1.00	1.01	1.04	1.00	1.01	1.12	1.05	1.01	1.00		
Shannon	0.0391	0.5587	0.0166	0.0860	0.0184	0.0329	0.1039	0.0017	0.0455	0.3076	0.1495	0.0386	0.0168		
Shannon true diversity	1.04	1.75	1.02	1.09	1.02	1.03	1.11	1.00	1.05	1.36	1.16	1.04	1.02		

*The sourdoughs are characterized by the identity of the baker and the bread-making practice.*

We first analyzed the alpha diversity (Tables 2, 4). The number of *Saccharomycetales* species ranged from 2 to 12 within sourdoughs. All sourdoughs but one (Sourdough 2) had a single dominant species (Tables 2, 4). The Simpson and Shannon indices were low (below 0.06 and 0.15, respectively) and the effective number of species was close to one for all sourdoughs except two (Table 4). These two sourdoughs had a higher diversity for different reasons. One (Sourdough 8) had a dominant species with a frequency of 0.94 and 11 other rare species (Table 4). The second (Sourdough 2) had two species with a frequency of 0.19 and 0.8 as well as three additional rare species (Table 4). The effective number of species estimated from the Simpson and Shannon indices were of 1,12 and 1,36 respectively for sourdough 8 and 1,49 and 1,75 for sourdough 2. There was no evidence of difference in alpha diversity between sourdoughs of the two groups of bakery practices (Table 4).

**FIGURE 3:**
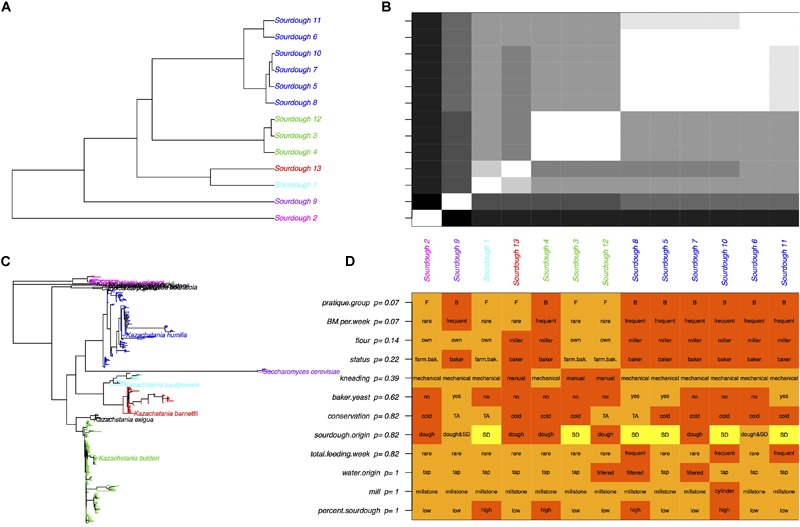
Yeast species diversity among sourdoughs and its link to bakery practices. **(A)** Tree of sourdough yeast communities based on the UniFrac distances. Colors correspond to the “yeasts’ community” group variable derived from a hierarchical clustering, **(B)** UniFrac distances between sourdoughs. White corresponds to close communities and black to the most distant communities. **(C)** Phylogenetic tree based on the OTU sequences. Colors correspond to the dominate species of each sourdough yeast community presented in **(A,B)**. **(D)** Baker practices and adjusted *p*-values obtained with an exact Fisher test between the practice and the “community” group. FDR correction was used to account for multiple testing.

Given that all sourdoughs but one were dominated by only one species, the weighted UniFrac distances between sourdoughs were strongly related to the phylogenetic distances between dominating species (Figure 3). The 13 sourdough communities were clustered into five groups: the five sourdoughs dominated by *K. humilis*, the three sourdoughs dominated by *K. bulderi*, the two sourdoughs dominated by *K. barnettii* or *K. saulgeensis* and the two groups represented by a sourdough dominated by either *K. unispora* or *S. cerevisiae*. These groups did not cluster sourdoughs according to their geographical origin.

Overall, we did not found a significant correlation between the UniFrac distances and the distances between bakers’ practices estimated by their difference in axis 1 coordinates on the MCA (Mantel test *p* = 0.19). Due to the small sample size and the strong dependence between practices variables, we could not select one or several specific practices variables that would explain the yeasts diversity of sourdough communities (Fisher exact tests, Figure 3). Instead, we examined the link between the sourdough community groups (five groups described above and Figure 3) and the practice group (bakers-like versus farmer-bakers like practices). The UniFrac distance differed between practice groups (*p* = 0.007) and these groups were not independent from the sourdough community groups (Fisher exact test, *p* = 0.01). A majority of sourdoughs collected from sourdough made with bakers-like practice (6 out of 8) were dominated by *K. humilis*, whereas none of the sourdoughs sampled from farmer-bakers practices’ bakeries were (Fisher exact text, *p* = 0.07*).* However, all sourdoughs but one (Sourdough 13) had a few reads identified as *K. humilis*. A part from the strong presence of *K. humilis* in sourdoughs from bakers but not from farmer-bakers, we were not able to distinguish species patterns related to specific practices.

**FIGURE 4:**
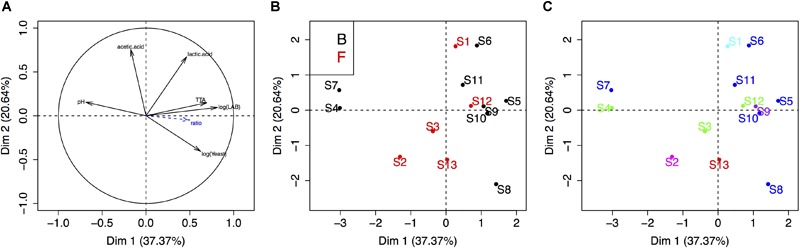
Principal component analysis of the sourdough’s biochemical and microbial properties. **(A)** Correlation circle of sourdough properties (acidity, population densities, etc.). **(B,C)** First two axis of the PCA analysis showing the distribution of sourdoughs according to the “practice” group (B, Bakers and F, farmer-baker) or the “yeast community” group (dark blue, sourdough dominated by *K. humilis*; green, by *K. bulderi*; red, by *K. barnettii*; purple, by *S. cerevisiae*; light blue, by *K. saulgeensis;* pink, by *K. unispora* and a yet undescribed *Kazachstania* species, see Figure 3).

Finally, we carried out a PCA to visualize the link between sourdough acidity (pH, TTA, lactic acid, acetic acid), yeast and bacteria density, bakery practice group and the five UniFrac groups of sourdoughs (Figure 4). The physico-chemical characteristics of these sourdoughs were previously reported in Lhomme et al. (2015b). The first two components explained 58% of the variation. The first axis was mostly explained by the variation in microbial density, pH and TTA, while the second axis reflected variation in lactic and acetic acid (Figure 4A). There was no clustering according to the bakery practice group (Figure 4B) or the yeast species composition of the sourdough (Figure 4C).

### Pattern of Yeast and Bacterial Species Diversity

The bacterial species diversity of these 14 sourdoughs have been analyzed previously (Lhomme et al., 2015b). The published data on the culture-based analysis of bacteria are presented Table 2. All sourdoughs contained *Lactobacillus sanfranciscensis*. This species was dominant (Freq > 0.5) in all sourdoughs but two when analyzed by culture-based method and dominant in all sourdoughs when analyzed by 16S metabarcoding (Lhomme et al., 2015b). The lack of diversity for the dominant LAB species limited any analysis of yeast-bacteria co-occurrence in this study. It also limits the testing of any link between metabolic properties and LAB species content. Four sourdoughs had more than one bacterial species, as detected by culture-based method. These sourdoughs (B3, B4, B5, B9) did not appear to have specific metabolic and microbial density properties. Indeed, they were not distributed on specific positions on the PCA compared to the ones where only *L. sanfranciscensis* was isolated (Figure 4).

## Discussion

In this work, we analyzed the yeast diversity in a collection of 14 sourdoughs collected from bakers with different bread-making practices. We developed a metabarcoding method for analyzing fungal communities in bread sourdough. From our knowledge only two studies have used so far metabarcoding to characterize fungi in sourdoughs made of cereals (Minervini et al., 2015; Raimondi et al., 2017, see review in Weckx et al., 2018). They used regions of the LSU rDNA as barcode. We used ITS1 that has been shown to better discriminate closely-related yeast species (Vu et al., 2016). We tested our method on a synthetic sourdough composed of flour and known sourdough yeast species in equal proportion. After data analysis of this synthetic sourdough, all the added yeast species were recovered showing that ITS1 barcode allows the discrimination of these yeast species. However, the species were detected in different proportions, revealing the limit of quantitative analysis using the pyrosequencing approach. The added species were found in low frequencies (9.4% of the total sourdough’s reads). The low representation of *Saccharomycetales* in the synthetic sourdough could be related to a DNA extraction, PCR or amplicon sequencing problem. The high proportion of non-*Saccharomycetales* reads also revealed the natural flour fungal composition. When analyzing the fungal distribution in natural sourdoughs, the *Saccharomycetales* were dominant compared to the non–*Saccharomycetales* species. In natural sourdough, where fermentation has occurred, the density of non-fermentative fungi, such as wheat pathogens, may be reduced by the production of metabolic inhibitors such as alcohol, acids and carbon dioxide during the fermentation process (Caplice and Fitzgerald, 1999).

By adding sourdough strain sequences to the public database, we improved the rate of OTU assignment at the species level. However, the “UNITE completed” database was not sufficient to assign all OTU reads at the species level. A total of 79.6% of the total number of reads were assigned to 60 species. The other reads were not assigned at species level. This might be related to a lack of power of ITS1 to discriminate species. It might also be related to a lack of reference sequences in databases. Different databases exist to assign fungal rDNA regions: INSD (international nucleotide sequence database), GenBank (Benson et al., 2006), ENA, DDBJ (Nakamura et al., 2013), SILVA (Pruesse et al., 2007), Ribosomal Database Project II (Cole et al., 2014), FUSARIUM-ID (Geiser et al., 2004), YEASTIP (Weiss et al., 2013) and UNITE databases. We chose the UNITE database because it is a commonly used and curated database for fungal barcoding. In addition, it contained non-translated parts of the rDNA region (ITS1 -5.8S- ITS2, Kõljalg et al., 2013). The presence of SSU parts in the reference database may alter assignments in favor of sequences containing SSU parts. However, the UNITE database still needs to be improved. For instance, taxonomical definitions need to be modified for “*Candida*” or “*Incertae sedis*”. More ITS1 sequences still need to be completed to contain sequences corresponding to all of the 100,000 described fungal species (Ainsworth, 2008). It would be particularly easy to include the 1100 known species of *Saccharomycetales* (432 in our completed version).

Culture-based and non-culture-based approaches gave similar results for the dominant species except for one species, the not-yet described *Kazachstania* species also discovered in boza from Bulgaria (Gouliamova et al., unpublished), and identified as *K. exigua* by the metabarcoding method. We did not add its ITS1 sequence to complete the UNITE database since the species was not described yet. The ITS1 sequence of this species and *K. exigua* are close, explaining the assignment to *K. exigua*. Strains of this species were previously identified as *K. exigua* by a previous publication that used a region of the LSU as barcode (Gouliamova et al., 2012). The *K. exigua* species has been described in many different sourdoughs (De Vuyst et al., 2016; Carbonetto et al., 2018). Based on the partial sequence of the large rDNA subunit (D1D2) and based on the ITS1 sequence, it clustered with this new species, as well as with the recently described species *K. saulgeensis* (Jacques et al., 2016; Carbonetto et al., 2018). Additional studies are needed to shed light on this closely related complex of yeast species.

As found previously, a single yeast species dominated the fungal community in each sourdough. As expected in fermented products (Lv et al., 2013; Illeghems et al., 2012; Tamang et al., 2016), *Saccharomycetales* species dominated in all the natural sourdoughs with the exception of Sourdoughs 9 and 14, which had a lower number of reads. Interestingly, the most commonly known bakery yeast species, *S. cerevisiae*, was not the major species, and no other species of the *Saccharomyces sensus stricto* clade was found. Only four of the participating bakers used commercial baker’s yeast. This might explain the overall low occurrence of *S. cerevisiae*. For three of them, *S. cerevisiae* was not isolated from their sourdough. These bakers used as chief sourdough, a piece of final sourdough of the previous bread-making process. This piece is collected before kneading, the step during which commercial yeast is usualy added. Therefore, our results suggest that commercial yeast does not invade the sourdough when used for other purposes in the bakeries. This finding should be confirmed on a higher number of samples or with dedicated experiments.

Most of the sourdoughs’ yeast species belonged to the *Kazachstania* clade. Overall, the *Kazachstania* species represented 91% of the 519 strains and 65.59% of the reads from natural sourdoughs. Seven *Kazachstania* species were detected (*K. humilis, K. bulderi, K. barnettii, K. unispora, K. saulgeensis, K. servazzii* and an as-yet undescribed *Kazachstania* sp), including a recently isolated species, *K. saulgeensis* (Jacques et al., 2016). The *Kazachstania humilis* and *K. exigua* species have been detected in sourdoughs in many different studies (De Vuyst et al., 2016; Carbonetto et al., 2018). The other ones are less commonly found.

Using the beta diversity index, we classified sourdoughs into five groups. The first group was characterized by the presence of *K. humilis* as the dominant species. The second group contained sourdoughs with *K. bulderi* as the dominant species. The other groups included the remaining species. The sourdoughs made by bakers harbored mostly *K. humilis* but also, in one case, *S. cerevisiae* or *K. bulderi*. The sourdoughs made by farmer-baker practices harbored a higher *Kazachstania* species diversity (*K. bulderi, K. unispora, K. saulgeensis, K. barnettii*, and the as-yet undescribed *Kazachstania* sp). These results highlight the importance of conserving diverse bakery practices to maintain yeast species diversity. However, additional sourdoughs should be analyzed to confirm this finding and understand if they are specific practices which drive the diversity and structure of these sourdough microbial communities.

No geographical structuration of the distribution of the dominant yeast species was found in our French sample. This could be due to a lack of statistical power given the small size of our sample. Numerous studies have been carried out on wheat sourdoughs’ microbial diversity in Europe, especially in Belgium and Italy. It was found that *S. cerevisiae* was the most frequent dominant yeast species, followed by *Wickerhamomyces anomalus* and *K. humilis* in Belgium and Italy, respectively (for a review, see De Vuyst et al., 2016; Carbonetto et al., 2018). In our French samples, we found that *K. humilis* was the most widespread dominant yeast species, followed by *K. bulderi*. We did not detect *W. anomalus*. Overall, the French sourdoughs studied here harbored higher species richness than Italian and Belgium sourdoughs. A previous study on 16 French sourdoughs collected in non-organic bakeries showed that all but one sourdough contained *S. cerevisiae* as the dominant species (Lhomme et al., 2015a). Our study reveals a much higher yeast species diversity. This result contrasted with the one on bacteria. Indeed, the *L. sanfranciscensis* species was the dominant bacterial species in 41 out of 45 French sourdoughs (Lhomme et al., 2015a,b, 2016; Michel et al., 2016). The origin of the yeast species diversity is still unknown. The drivers for the establishment and composition of LAB sourdough diversity have been reviewed (Gobbetti et al., 2016; Gänzle and Ripari, 2016). Numerous factors drive the LAB species diversity, such as the house microbiota, the flour species, and other ingredients. Drift has been shown to drive change in sourdough bacterial species in laboratory experiments (Lhomme et al., 2014; Rizzello et al., 2015). Several studies revealed that dispersion from the bakery environment or from the flour may also explained part of the composition of the sourdough bacterial community (Minervini et al., 2015; Minervini et al., 2018). Moreover, differences in sourdough environment, such as pH, cereal composition of the flour or fermentation time, may drive sourdough microbial community divergence (see for a review Gänzle and Ripari, 2016). The factors that drive yeast species diversity have been less explored. Additional studies, including a wider collection of sourdoughs or experimentations on different bakery practices, need to be carried out to better understand the origin and conservation of yeast species and genetic diversity.

## Author Contributions

CU carried out the experiments, analyzed all the data, and drafted the manuscript. PM analyzed the pyrosequencing data. SC analyzed the new species. JL and DS designed the project, analyzed all the data and revised the manuscript.

## Conflict of Interest Statement

The authors declare that the research was conducted in the absence of any commercial or financial relationships that could be construed as a potential conflict of interest.

## Footnotes

1 http://genome.jouy.inra.fr/yeastip/

2 http://www.eurofins.com/en.aspx

3 https://unite.ut.ee/sh_files/UNITE_public_01.08.2015.fasta.zip
