# Supplementary material for: Fungal Species Diversity in French Bread Sourdoughs Made of Organic Wheat Flour
Source: Front Microbiol. 2019 Feb 18;10:201. doi: 10.3389/fmicb.2019.00201 (PMC6387954; doi:10.3389/fmicb.2019.00201)
Supplement: Supplementary file 1 [file Table_1.DOCX]

**Table S1:** Sequences of oligonucleotide primers used for PCR amplification and sequencing for culture-based identification

| Primer set | Oligonucleotide sequence (5' --> 3') | Sequence length (bp) | Target gene | | Reference |
| --- | --- | --- | --- | --- | --- |
| SR21 | CTTAATCTTTGAGACAAGC | 2540 | intergenic spacer rDNA (NTS) | | Nguyen *et al.*, 2009 |
| LR13 | CGATCTGCTGAGATTAAG |  |  |  |  |
| NL1 | GCATATCAATAAGCGGAGGAAAAG | 550 | 26S gene D1/D2 region ribosomal gene | | O'Donnell, 1993 |
| NL4 | GGTCCGTGTTTCAAGACGG |  |  |  |  |
| CA5R | GTGAACAATGGATGGACCAGATTCGTCG | 800 | *act1* gene | | Kan, 1993 |
| CA14 | AACTGGGATGACATGGAGAAGATCTGGC |  |  |  |  |
| 58A2R | CTGCGTTCTTCATCGAT | 375-650 | Internal transcribed spacer (ITS1) | Martin & Rygiewicz, 2005 | |
| NSA3 | AAACTCTGTCGTGCTGGGGATA |  |  |  |  |
