# Supplementary material for: Fungal Species Diversity in French Bread Sourdoughs Made of Organic Wheat Flour
Source: Front Microbiol. 2019 Feb 18;10:201. doi: 10.3389/fmicb.2019.00201 (PMC6387954; doi:10.3389/fmicb.2019.00201)
Supplement: Supplementary file 2 [file Table_2.docx]

**Table S2:** List of strains used to set up the meta-barcoding experiment.

| **Species** | **Strain ID** | **Strains mixed in the synthetic sourdough** | **ITS sequences used for** *in silico* **selection of fungal specific primers** | **DNA used to test fungal specific primer pairs** | **Sanger sequences added to the Unite database** |
| --- | --- | --- | --- | --- | --- |
| *Zea mays* | B73 line |  |  | x |  |
| *Triticum aestivum* | Courtot variety |  |  | x |  |
| *Nigella damascena* |  |  |  | x |  |
| *Candida carpophila* | B1_TP_21 | x | x | x | x |
| *Kazachstania humilis* | B5r_TP1_1 | x | x | x | x |
| *Kazachstania humilis* | B5r_TP1_13 |  | x | x |  |
| *Hyphopicchia pseudoburtonii* | B1_TP_40 | x | x | x | x |
| *Hyphopicchia pseudoburtonii* | CBS_2455a |  |  | x |  |
| *Kazachstania bulderi* | B3_LC1_1 |  | x | x |  |
| *Kazachstania bulderi* | B3_TP1_1 | x | x | x |  |
| *Kazachstania saulgeensis* | B1r_TP1_1 | x | x | x | x |
| *Kazachstania unispora* | B2_AC_10 | x | x | x |  |
| *Kazachstania unispora* | B2r_TP1_1 |  | x | x | x |
| *Kazachstania unispora* | B2r_TP1_13 |  | x | x |  |
| *Rhodotorula mucilaginosa* | B2_AC_31 |  | x | x |  |
| *Rhodotorula mucilaginosa* | B2_AC_37 |  | x | x |  |
| *Rhodotorula mucilaginosa* | B2_AC_33 | x |  |  |  |
| *Saccharomyces cerevisiae* | B1r_TP1_33 | x | x | x |  |
| *Saccharomyces cerevisiae* | B5r_TP2_12 |  | x | x |  |
| *Torulaspora delbrueckii* | B1_LC_1 | x | x | x |  |
| *Torulaspora delbrueckii* | B1_AC_26 |  | x | x |  |
| *Candida carpophila* | CLIB1330^T^ |  | x |  | x |
| *Candida glabrata* | CLIB298^T^ |  | x |  | x |
| *Candida parapsilosis* | CLIB214 ^T^ |  | x |  |  |
| *Hyphopichia burtonii* | CBS2352 ^T^ |  | x |  | x |
| *Kazachstania humilis* | CLIB1323^T^ |  | x |  | x |
| *Kazachstania barnettii* | CLIB433 |  | x |  |  |
| *Kazachstania bulderi* | CLIB596 ^T^ |  | x |  |  |
| *Kazachstania exigua* | CLIB179 ^NT^ |  | x |  |  |
| *Kazachstania unispora* | CLIB234 ^T^ |  | x |  |  |
| *Meyerozyma guilliermondii* | CLIB515 ^T^ |  | x |  | x |
| *Millerozyma farinosa* | CLIB517 ^T^ |  | x |  | x |
| *Pichia kudriavzevii* | CLIB884 ^T^ |  | x |  | x |
| *Pichia occidentalis* | CBS5459 |  | x |  |  |
| *Saccharomyces cerevisiae* | CLIB227 ^T^ |  | x |  |  |
| *Scheffersomyces stipitis* | CLIB187 ^T^ |  | x |  | x |
| *Torulaspora delbrueckii* | CLIB230 ^T^ |  | x |  |  |
| *Wickerhamomyces anomalus* | CLIB284 ^T^ |  | x |  |  |
