# Supplementary material for: Fungal Species Diversity in French Bread Sourdoughs Made of Organic Wheat Flour
Source: Front Microbiol. 2019 Feb 18;10:201. doi: 10.3389/fmicb.2019.00201 (PMC6387954; doi:10.3389/fmicb.2019.00201)
Supplement: Supplementary file 3 [file Table_3.DOCX]

**Table S3:** Tested fungi ITS primers, from 5’ to 3’. “_RC”= reverse complement.

| **Primer name** | **Primer sequence** | **Reference** |
| --- | --- | --- |
| ITS1F | CTTGGTCATTTAGAGGAAGTAA | Orgiazzi *et al.*, 2013 |
| ITS1-FL | CAAACTTGGTCATTTAGAGGAAGTAA | Taylor *et al.*, 2008 |
| ITS3 | GCATCGATGAAGAACGCAGC | Orgiazzi *et al.*, 2013 |
| ITS5 | TCCTCCGCTTATTGATATGC | Schoch *et al.*, 2012 |
| ITS2r_RC | GCATCGATGAAGAACGCAGC | Orgiazzi *et al.*, 2013 |
| ITS1F | CTTGGTCATTTAGAGGAACTAA | Gardes & Bruns, 1993 |
| ITS5f_RC | CCTTGTTACGACTTTTACTTCC | Bellemain *et al.*, 2010 |
| ITS1 | TCCGTAGGTGAACCTGCGG | Bellemain *et al.*, 2010 |
| ITS4r | GGAAGTAAAAGTCGTAACAAGG | Schoch *et al.*, 2012 |
| ITS4Br_RC | CTGGACCGTGTACAAGTCTCCTG | Bellemain *et al.*, 2010 |
| ITS4r | TCCTCCGCTTATTGATATGC | Bellemain *et al.*, 2010 |
| TW13r_RC | CGTCTTGAAACACGGACC | Taylor & Bruns, 1999 |
| TW13-64Pr_RC | CGTCTTGAAACACGGACCAAATGAACCACAAAGAAAC | Taylor *et al.*, 2008 |
| TW13-67Pr_RC | CGTCTTGAAACACGGACCAAATCGGCGGCTAAGAAAC | Taylor *et al.*, 2008 |
| TW13-102Pr_RC | CGTCTTGAAACACGGACCAAATAGTTACTAAAGAAAC | Taylor *et al.*, 2008 |
| TW13-126Pr_RC | CGTCTTGAAACACGGACCAACAGTGCTATGAAGAAAC | Taylor *et al.*, 2008 |
| TW14r | GCTATCCTGAGGGAAACTTC | Taylor & Bruns, 1999 |
| Ctb6f | GCATATCAATAAGCGGAGG | Taylor & Bruns, 1999 |
| Ctb6f_RC | CCTCCGCTTATTGATATGC | Taylor & Bruns, 1999 |
| ITS4r_RC | CCGTTCCTTGTCTATGTTCCTTG | Gardes & Bruns, 1993 |
| NSI1 | GATTGAATGGCTTAGTGAGC | Martin & Rygiewicz, 2005 |
| 58AIF | GCATCGATGAAGAACGC | Martin & Rygiewicz, 2005 |
| 58A2F | ATCGATGAAGAACGCAG | Martin & Rygiewicz, 2005 |
| NLB4_RC | GTCATAGAGGGTGAGAATCC | Martin & Rygiewicz, 2005 |
| 58A2R | CTGCGTTCTTCATCGAT | Martin & Rygiewicz, 2005 |
| ITS5 | GGAAGTAAAAGTCGTAACAAGG | Kurtzman & Robnett, 2003 |
| ITS4_RC | GCATATCAATAAGCGGAGGA | Kurtzman & Robnett, 2003 |
| NLC2 | GAGCTGCATTCCCAAACAACTC | Martin & Rygiewicz, 2005 |
| NSA3 | AAACTCTGTCGTGCTGGGGATA | Martin & Rygiewicz, 2005 |
