# Supplementary material for: Fungal Species Diversity in French Bread Sourdoughs Made of Organic Wheat Flour
Source: Front Microbiol. 2019 Feb 18;10:201. doi: 10.3389/fmicb.2019.00201 (PMC6387954; doi:10.3389/fmicb.2019.00201)
Supplement: Supplementary file 4 [file Table_4.docx]

**Table S4:** Index sequence for each sample.

| Sample ID | Nucleic Acid Conc. (ng/µl) | Index seq. |
| --- | --- | --- |
| SD_1 | 70,2 | ACGAGTGCGT |
| SD_2 | 61,7 | ACGCTCGACA |
| SD_3 | 66,8 | AGACGCACTC |
| SD_4 | 68,6 | AGCACTGTAG |
| SD_5 | 55,2 | ATCAGACACG |
| SD_6 | 59,6 | ATATCGCGAG |
| SD_7 | 58,7 | CGTGTCTCTA |
| SD_8 | 60 | CTCGCGTGTC |
| SD_9 | 56,9 | TCTCTATGCG |
| SD_10 | 65,4 | TGATACGTCT |
| SD_11 | 59 | CATAGTAGTG |
| SD_12 | 56,4 | CGAGAGATAC |
| SD_13 | 60,6 | ATACGACGTA |
| SD_14 | 58,8 | TCACGTACTA |
| SD_15 | 52,9 | CGTCTAGTAC |
