# Supplementary material for: Fungal Species Diversity in French Bread Sourdoughs Made of Organic Wheat Flour
Source: Front Microbiol. 2019 Feb 18;10:201. doi: 10.3389/fmicb.2019.00201 (PMC6387954; doi:10.3389/fmicb.2019.00201)
Supplement: Supplementary file 5 [file Table_5.DOCX]

**Table S5**: Sequences added to the UNITE data.
