# Supplementary material for: Fungal Species Diversity in French Bread Sourdoughs Made of Organic Wheat Flour
Source: Front Microbiol. 2019 Feb 18;10:201. doi: 10.3389/fmicb.2019.00201 (PMC6387954; doi:10.3389/fmicb.2019.00201)
Supplement: Supplementary file 6 [file Table_6.DOCX]

**Table S6.** Total number of reads per sourdough, number of reads and OTUs per sourdough when more than five reads belonged to the OTU

| Sample | Total read count | read count >5 | OTU count >5 |
| --- | --- | --- | --- |
| Sourdough_1 | 13794 | 13608 | 168 |
| Sourdough_2 | 11909 | 11645 | 225 |
| Sourdough_3 | 12498 | 12206 | 303 |
| Sourdough_4 | 13800 | 13430 | 331 |
| Sourdough_5 | 11113 | 11032 | 179 |
| Sourdough_6 | 19742 | 19569 | 156 |
| Sourdough_7 | 18758 | 18265 | 351 |
| Sourdough_8 | 17173 | 16924 | 348 |
| Sourdough_9 | 7371 | 7212 | 217 |
| Sourdough_10 | 13830 | 13723 | 104 |
| Sourdough_11 | 13399 | 13207 | 168 |
| Sourdough_12 | 13362 | 13113 | 303 |
| Sourdough_13 | 15960 | 15697 | 215 |
| Sourdough_14 | 13146 | 12724 | 239 |
| Synthetic sourdough | 10323 | 9870 | 320 |
| total | 206178 | 202225 |  |
