# Supplementary material for: Fungal Species Diversity in French Bread Sourdoughs Made of Organic Wheat Flour
Source: Front Microbiol. 2019 Feb 18;10:201. doi: 10.3389/fmicb.2019.00201 (PMC6387954; doi:10.3389/fmicb.2019.00201)
Supplement: Supplementary file 7 [file Table_7.DOCX]

Table S7. Read number assigned to the species level in each sourdough. SD = sourdough, Nat SD freq=overall natural sourdough
